# Design, randomization and methodology of the TriAtiva Program to reduce obesity in school children in Southern Brazil

**DOI:** 10.1186/s12889-015-1727-0

**Published:** 2015-04-11

**Authors:** Roberta R Friedrich, Lisandrea C Caetano, Mariana D Schiffner, Mário B Wagner, Ilaine Schuch

**Affiliations:** Graduate Program in Child and Adolescent Health, School of Medicine, Federal University of Rio Grande do Sul, Rio Grande do Sul, Brazil; Department of Nutrition, School of Medicine, Federal University of Rio Grande do Sul, R. Ramiro Barcelos, 2400, Bairro Santa Cecília, Porto Alegre, 90035003 Rio Grande do Sul Brazil; Departament of Nutrition, School of Medicine, Federal University of Rio Grande do Sul, R. Ramiro Barcelos, 2400, Bairro Santa Cecília, Porto Alegre, 90035003 Rio Grande do Sul Brazil; Graduate Program in Child and Adolescent Health, School of Medicine, Federal University of Rio Grande do Sul, R. Ramiro Barcelos, 2400, Bairro Santa Cecília, Porto Alegre, 90035003 Rio Grande do Sul Brazil; Department of Nutrition, School of Medicine, Federal University of Rio Grande do Sul, Brazil. Centre of Food and Nutrition Studies (CESAN) Clinical Hospital of Porto Alegre, Porto Alegre, 90035003 Rio Grande do Sul Brazil

**Keywords:** Obesity, Prevention & control, Nutrition education, Physical education and training, Weight reduction programmes, Schools, Randomized controlled clinical trial

## Abstract

**Background:**

The prevalence of child obesity in Brazil has increased rapidly in recent decades. There is, therefore, an urgent need to develop effective strategies to prevent and control child obesity. In light of these considerations, an intervention program with a focus on nutrition education and physical activity was developed for to prevent and control obesity in schools. The intervention was called the TriAtiva Program: Education, Nutrition and Physical Activity. This article describes the design, randomization and method used to evaluate the TriAtiva program.

**Methods/design:**

This randomized controlled cluster trial was performed in 12 municipal schools in the city of Porto Alegre/RS (six schools in the intervention group and six control schools) which offered first- through fourth grade, during one school year. The TriAtiva Program was implemented through educational activities related to healthy eating and physical activity, creating an environment which promoted student health while involving the school community and student families. The primary outcome of the present study was body mass, while its secondary outcomes were waist circumference, percent body fat, blood pressure and behavioural variables such as eating habits and physical activity levels, as well as the prevalence, incidence and remission rates of obesity.

**Discussion:**

The intervention was developed based on a comprehensive review of controlled trials of similar design. The TriAtiva Program: Education, Nutrition and Physical Activity was the first study in Southern Brazil to use a randomized controlled design to evaluate an intervention involving both nutrition education and physical activity in schools. Our results will contribute to the development of future interventions aimed at preventing and controlling child obesity in schools, especially in Brazil. Brazilian Clinical Trials Registry (REBEC) number RBR2xx2z4.

## Background

The prevalence of child obesity has increased significantly in both developed and developing countries, resulting in a severe impact on child and adolescent health [1-9]. A recent representative study of nutritional status in Brazil - namely, the 2008–2009 Family Budget Survey (POF) - found the prevalence of overweight in 5- to 9-year-old children to be between 32 and 40% in the Southeast, South and Central-West regions of the country, and 25 to 30% in the North and Northeast regions. This age range also saw the greatest increase in obesity rates during the time period studied [10]. Regarding student populations, the National Student Survey (PENSE) has found the prevalence of overweight to be of 23% in primary school students in Brazil [11].

Recent transformations in Brazil caused by increasing modernization and urbanization, have led to negative lifestyle changes such as inadequate diets, an increased intake of ultra-processed foods and decreased consumption of fruits and vegetables, and sedentary habits such as an increase in the time spent watching television and playing videogames combined with a decrease in physical activity, all of which have contributed to the increased prevalence of childhood overweight and obesity [12-14].

Children are a priority population for implementing prevention strategies against obesity and related chronic diseases [15]. Since lifestyle habits at this age are not yet fully established, they are more susceptible to change as a result of preventive interventions than the habits of adult individuals.

Schools are therefore a privileged setting for initiatives targeting the improvement of health and nutritional status in children, and a strategic location for the implementation of health promotion programmes [16].

Although there is no consensus as to which interventions are most effective against obesity, most of these strategies focus on lifestyle changes, nutritional counselling and physical activity [17]. The combination of physical activity and nutrition education is more effective than each of these measures individually in decreasing body mass index (BMI), as well as preventing and controlling obesity in students [18].

The rising obesity rates in Brazil have led to the implementation of public policies regarding health promotion programs, healthy eating and physical activity in across several settings, including schools [19]. However, few studies have used a well-designed methodology to develop lifestyle intervention programs for school-aged children in Brazil [20-24].

The aim of the present study was to assess the effects of an intervention program involving nutrition education and physical activity - namely, the TriAtiva Program: education, nutrition and physical activity - on the prevention and control of obesity in public primary school children in the city of Porto Alegre/Brazil using a randomized controlled trial.

## Method

### Study design

The TriAtiva Program was evaluated in a randomized, controlled cluster trial performed in public schools of Porto Alegre, Rio Grande do Sul, Brazil, which served first- to fourth-grade students. Schools were randomly assigned to the intervention or control condition to avoid contamination between groups. The program was implemented during the 2013 school year. This study was approved by the Research Ethics Committee of the Clinical Hospital of Porto Alegre/RS (n° 215.661/CAAE 12406713.6.0000.5327) and entered in the Brazilian Clinical Trials Registry (REBEC) under number RBR2xx2z4. This study is in the data analysis phase.

### Eligibility, randomization and blinding

All children of both genders enrolled in first- through fourth-grade who attended afternoon classes in primary municipal schools in the city of Porto Alegre/RS were eligible for participation in the present study.

Students who were unable to undergo anthropometric assessment, who had special needs, or were absent from school on the two occasions on which anthropometric measurements were taken were excluded from the study. Students who, despite parental consent, refused to allow anthropometric measurements to be taken and to participate in intervention activities at the beginning of the study were also excluded from the sample.

Of the 50 primary schools assessed for eligibility in the city of Porto Alegre/RS, 16 were excluded from the study. Fourteen of these were excluded for not offering afternoon classes for all grades from first through fourth, one for being a night school, and the other for catering exclusively to special needs students. As a result, 34 schools remained eligible for cluster randomization.

To reach the required number of students (assuming a mean of 22.4 students per classroom based on the list of students enrolled in municipal schools provided by the Board of Education of Porto Alegre/RS), a total of 12 schools would need to be included in the randomization procedure. Six of these would be assigned to the intervention group, while the remaining six would compose the control group.

**Figure 1:**
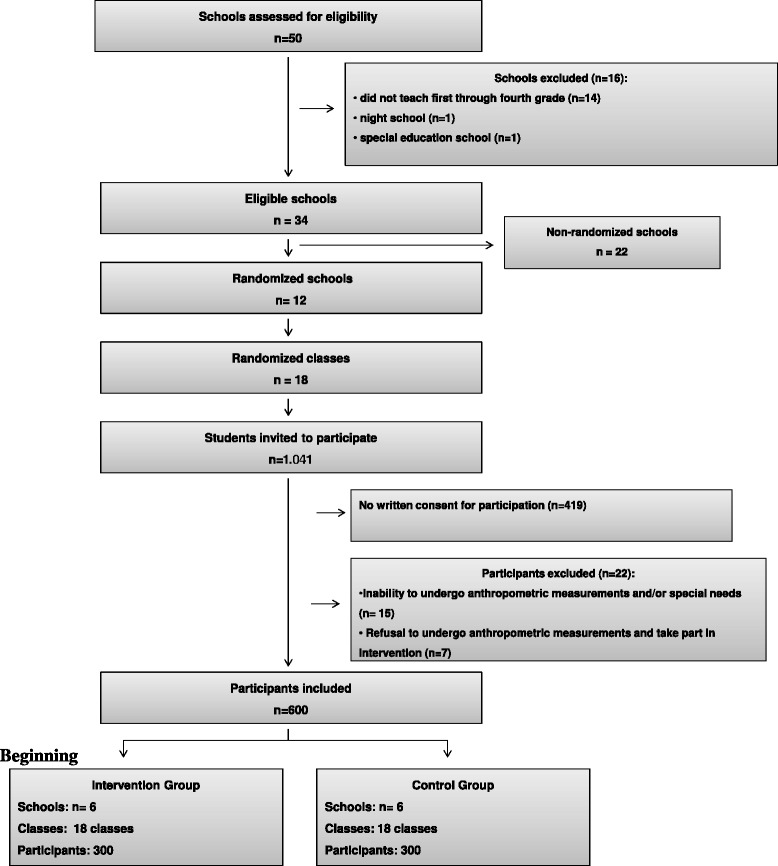
Flowchart of schools and participants.

Schools were allocated between groups using the cluster randomization function of the *WinPepi Software*. After all 12 schools were randomized (six to the control and six to the intervention group), random sampling was once again used to select the participating classes in each institution. Three classes from grades first through fourth were selected in each school, for a total of 36 participating classes. The final sample consisted of 600 students. A flowchart of the study is shown in Figure 1.

To avoid contamination between groups, schools were randomly assigned to the intervention or control groups, and only the participants were blinded. Additionally, the teachers and parents of students in the intervention group were told that the program aimed to promote student health, but were not informed as to the main goal of the study.

### Intervention

The TriAtiva Program: Education, Nutrition and Physical Activity, was developed following a comprehensive review of randomized controlled trials of lifestyle interventions aiming to prevent and control obesity in student populations by promoting nutrition education and physical activity [18] .

The TriAtiva Program: Education, Nutrition and Physical Activity was a year-long initiative which aimed to implement educational activities about healthy eating and physical activity so as to develop a favourable environment for student health. The nutrition education and physical activity interventions involved in the program were developed and implemented by a research team of nutritionists, physical education teachers and professors of nutrition and physical education. All interventions were adapted to each school year based on a participative and playful approach.

TriAtiva Program activities were performed every 15 days during school hours, and physical activity interventions were alternated with nutrition education initiatives, for a total of six months of activities. At the end of each TriAtiva event at school, extracurricular activities related to the topic of discussion were planned.

The nutrition education intervention was centred on healthy eating and related topics, such as food groups, the origin and production of different food items, and the selection of healthy foods. The aim of these activities was to promote healthy eating by increasing fruit and vegetable intake, reducing the consumption of processed and highly caloric foods and drinks, and increasing water intake. To encourage water intake, each student was given a bottle.

To encourage the consumption of the school meals offered by the National Student Nutrition Program (NSNP) and of healthy foods and drinks brought from home, a competition was set up. Students who ate their meals at school or brought healthy snacks from their homes received raffle tickets, and the winner of each class received a medal, a soccer ball and a certificate of participation. The school menu was planned by government nutritionists, and school cafeterias were run in accordance with Municipal Law (n° 10.167) [25].

The physical education program was based on a Developmental Physical Activity approach, and implemented through outdoor recreational activities which aimed to improve interpersonal relationships, group work, motor skills, aerobic fitness, coordination and agility, and encourage self-expression through dance and music. Children were also encouraged to substitute activities such as watching television, using the computer or playing videogames by more active leisure-time pursuits.

Recreational activities based on popular games were also implemented, such as hopscotch, potato-sack races, and Simon Says, all of which were performed in combination with the nutrition education intervention. The intervention group also followed the regular physical education curriculum, which included two 50-minute physical education classes a week. The topics discussed in the TriAtiva Program and the objective of each initiative are explained in greater detail in Table 1.

**Table 1:** **Educational activities in the TriAtiva Program**

**Topics discussed in the TriAtiva Program**	**Objective**
**•** Nutrition Education	
**What do we eat and why?**	To emphasize the importance of eating a variety of foods and discuss different methods of food preparation. To provide information about the importance of healthy eating habits in childhood for growth and development, using child-appropriate language.
**Which foods are healthy?**	To encourage the student to reflect on the importance of taking healthy snacks to school and eating healthy at school. To consolidate knowledge regarding healthy eating by assembling one’s own lunchbox.
**The origin of food**	To study the origin of different food items and distinguish between animal and plant products. To identify foods and their derivatives.
**Food groups**	To study the food groups through a food circle, games and teamwork.
**Recognizing foods using all five senses.**	To work on perceiving and recognizing food items through the five senses (touch, smell, hearing, taste and sight).
**Importance of water**	To stimulate water intake and discuss its importance to health by giving each student a water bottle.
**Topics discussed in the TriAtiva Program**	**Objective**
**•** Physical Activity	
**The importance of physical activity for health**	To showing the benefits of sports such as football, basketball, volleyball and swimming. To explain that sports improve motor coordination, physical conditioning and cognitive ability, and the ability to modify/create new strategies or methods to carry out different activities.
**Recreational activities performed individually or in pairs**	To stimulate motor skills, aerobic activities and coordination, while remembering facts about fruits, vegetables and sports.
**Recreational sport activities**	To stimulate motor skills, aerobic activity and coordination.
**Dance Activities (rhythms)**	To stimulate self-expression through music.
**Cooperative Activities**	To stimulate collective creativity.
**Challenge to reduce sedentarism**	To encourage children to substitute time spent watching television, playing videogames and using the computer for more active leisure habits. Children were motivated to have less than two hours of screen time for a week.

Parental involvement in the intervention was encouraged through meetings in which the intervention program and the initial results of the study were presented, and topics such as the importance of healthy eating and regular physical activity were discussed. Parents were also motivated to modify their own eating habits in addition to their children’s, and to increase their physical activity levels. Two meetings were held per year in each school to stimulate parental participation and give parents the opportunity to ask any questions they may have regarding the health of their children.

Teachers were also encouraged to develop activities centred on nutrition and physical activity, and invited to take part in program activities during their implementation by the research group.

**Figure 2:**
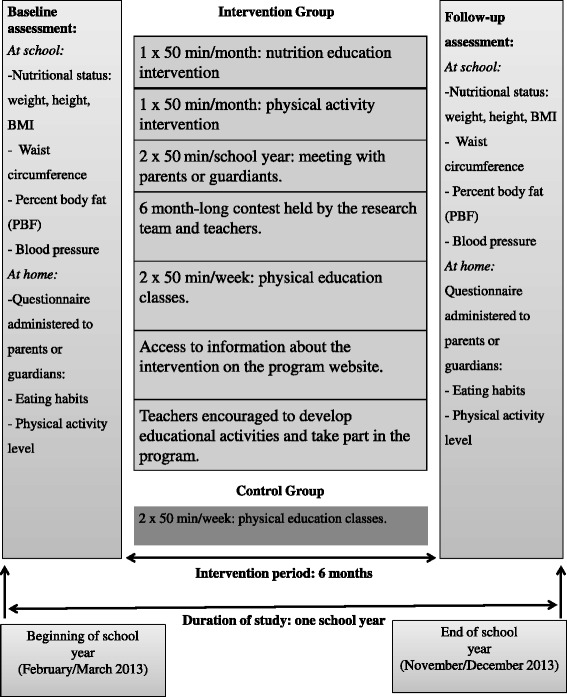
Description of the study and of the TriAtiva Program.

The school community and parents were also stimulated to seek further information on the program website. The present study and the TriAtiva Program are described in Figure 2.

### Control group

The control group did not receive any interventions, and followed the regular curriculum, which offered twice weekly physical education classes of 50 minutes each.

### Outcome measurements

The primary outcome of the present study was body mass, while its secondary outcomes were waist circumference, percent body fat, blood pressure and behavioural variables such as eating habits and physical activity levels, as well as the prevalence, incidence and remission rates of obesity.

Outcomes were measured at baseline and immediately after the intervention. To minimize sample loss, anthropometric measurements were taken on two different occasions in each school at both time-points . All researchers were trained prior to data collection, but were not blinded to participant allocation. Blood pressure and all anthropometric measurements (weight, height and waist circumference), save for percent body fat, were taken in duplicate.

### Weight, height and body mass index

Body mass index was defined as the ratio of weight in kg to height in meters squared (kg/m^2^). Weight and height were measured using calibrated equipment according to standard World Health Organization guidelines [26].

Weight was measured using a portable electronic scale, with a maximum capacity of 200 kg and a precision of 50 g (*Marte®* , PP200 Model). Students were weighed barefoot and wearing light clothing. Height measurements were taken using an *AlturaExata®* portable stadiometer measuring up to 2 with an accuracy of 1 mm. Participants were measured barefoot, with feet in parallel and ankles together, standing upright with their head in the Frankfurt Plane. If the two height measurements differed by more than 0.5 cm, a third measurement was taken.

Nutritional status was assessed based on age and gender norms for BMI Z-scores, which were calculated using the WHO criteria [26] employed by the Brazilian Ministry of Health, and the *Anthro Plus*® software.

### Waist circumference and percent body fat

Waist circumference (WC) was measured using a *Cescorf*® inelastic measuring tape accurate to 1 mm. Measurements were taken at the navel [27] and classified according to the criteria suggested by Taylor [28].

Percent body fat was assessed by bioelectrical impedance (BEI), using a *Biodynamics ®* analyser, model 450. These measures were classified using the cut-offs provided by Slaughter & Lohman [29].

### Blood pressure

Blood pressure was measured according to the VI Brazilian Arterial Hypertension Guidelines [30], using an *Omron*® sphygmomanometer (model HEM 705-CP). Blood pressure measurements were interpreted and classified using the age, gender and height norms provided by the *National High Blood Pressure Education Program Working Group on High Blood Pressure in Children and Adolescents* [31].

### Nutrition

Student nutrition was evaluated using an Eating Habits Questionnaire developed for subjects aged 5 years or older by the Food and Nutrition Surveillance System (SISVAN) and the National Ministry of Health. This instrument was administered to participant parents or guardians. The aim of the questionnaire is to verify the frequency with which the subject consumed a series of foods or beverages in the previous seven days. Some of these items are associated with a healthy diet (e.g. daily intake of beans, fruits, vegetables and milk or yoghurt), while others are associated with an unhealthy one (e.g. frequent intake of fried foods and sweets) [32]. The family intake of salt, sugar and oils was evaluated using open questions. The frequency with which students ate while watching TV or at school was also evaluated.

### Physical activity and sedentarism

Physical activity was assessed using the *Physical Activity Questionnaire for Older Children* (PAQ-C) [33], which was administered to parents or guardians. The PAQ-C evaluates moderate to intense physical activities performed in the previous seven days (including the weekend). The instrument contains nine questions about sports, games, physical activity at school and leisure habits. Each question is rated on a scale of 1 (did not engage in activity) to 5 (engaged in activity every day of the week). The final score is then classified into one of the following intervals: 1 - very sedentary; 2 - sedentary; 3 - moderately active; 4 - active; 5 - very active. The PAQ-C also includes a question about time spent watching television, two questions about the respondent’s physical activity level as compared to that of gender- and age-matched peers, and an additional question about illnesses which may have prevented the child from engaging in physical activity in the previous week. Sedentary behaviour was evaluated in terms of the amount of time spent watching television, playing videogames or on the computer.

### Socioeconomic status

The socioeconomic characteristics of student families was assessed using the Brazilian Economic Classification Criteria (CCEB) developed by the Brazilian Association of Research Companies (ABEP), which was used to classify families according to their socioeconomic status [34]. The CCEB classifies respondents into one of five economic levels ranging from A to E based on their ownership of household appliances, the employment of paid domestic help, and the educational level of the head of the family. This classification also allows for an estimation of monthly family income (R$).

### Prevalence, incidence and remission of obesity

Obesity was defined as a BMI Z score ≥ +2. The incidence of obesity was calculated as the percentage of students who were not obese at the beginning of the study, but who had become so by the end of the study period. Prevalence was defined as the number of individuals who were obese at the time of the study, and remission was calculated as the percentage of subjects who were obese at the beginning but not at the end of the study.

### Sample size calculation

The sample size of the present study was calculated based on a meta-analysis published by Friedrich et al. [18], which evaluated the effect of interventions involving both nutritional education and physical activity on the BMI of school-aged children and adolescents. Sample size was calculated using the *Power and Sample Size*® software. A sample size of 105 students per group would be required to detect a standardized difference of 0.45 in mean BMI (E/S = 0.45) with a significance of 5% and a statistical power of 90%. To correct for an estimated sample loss of 15% (F = 15/85 + 1) and the effects of the cluster design (2.0), the sample was increased to 250 per groups (500 in total).

### Data analysis

Data were entered into the *EpiData*® software, version 3.1, in duplicate to ensure the consistency of the results. Continuous variables were summarized as means and standard deviations, and categorical ones as absolute and relative frequencies. Continuous and categorical variables assessed at baseline were compared between groups using mixed model analysis or generalized estimating equations, respectively. Analyses were based on intention to treat. All analyses used to estimate the effect size of the intervention on continuous outcomes were presented as Z score units. Effect size at the end of the study will be estimated using a mixed model analysis of Z-scores, with sex and age as fixed factors, school as a random factor, and baseline Z-scores as a covariate. For continuous outcomes, the effect size was defined as the difference between the z-scores of the intervention and control groups at the end of the study, after adjusting for sex, age, and baseline values. Effect size was assessed based on the difference between standardized means, and interpreted according to Cohen’s guidelines [35]. The intraclass correlation coefficient (ICC) was used to compare the variability of measurements between groups. The effect of the intervention on categorical outcome measures was evaluated using a generalized estimating equation, adjusted for sex, age and school. Changes in the remission, incidence and prevalence rates of obesity in the intervention and control groups at the end of the study were also assessed. These values were presented as odds ratios adjusted for sex, age and baseline measures, and analysed using generalized estimating equations.

Data were analysed using the *Statistical Package for the Social Sciences* (SPSS)® for Windows, version 22.0.

## Discussion

The rapid economic growth and lifestyle changes which have recently taken place in Brazil coincided with a notable increase in the prevalence of overweight and obesity in children. Interventions aimed at preventing and controlling obesity have therefore gained increasing importance, especially in child populations, since lifestyle habits are not yet completely consolidated in this age group. To prevent obesity, there is a need to balance calorie intake and expenditure, which can be achieved by keeping healthy eating habits or performing regular physical activity. Therefore, the TriAtiva Program: Education, Nutrition and Physical Activity aimed to implement educational activities centred on healthy eating and physical activity to create an environment which would promote child health.

The TriAtiva Program: Education, Nutrition and Physical Activity consisted of the first randomized controlled trial of an intervention which used both nutrition education and physical activity to prevent obesity in school children. The results of this study will provide important evidence of the viability of the TriAtiva program in preventing obesity in school age children, and yield valuable information to other research groups which may wish to develop strategies to prevent child obesity through interventions involving nutritional education and physical activity in schools.
